# Moving towards Universal Health Coverage through the Development of Integrated Service Delivery Packages for Primary Health Care in the Solomon Islands

**DOI:** 10.5334/ijic.2447

**Published:** 2016-03-30

**Authors:** Stephen Whiting, Tenneth Dalipanda, Sjoerd Postma, Ayesha Jamshaid de Lorenzo, Audrey Aumua

**Affiliations:** 1 Consultant Health Service Delivery, WHO, Geneva, Switzerland; 2 Permanent Secretary for the Solomon Islands Ministry of Health and Medical Services, Honiara, Solomon Islands; 3 Chief of Party, Collaborative Support for Health Program, USAID/Country Lead, Management Sciences for Health, Monrovia, Liberia; 4 Technical Officer Health Systems, WHO Western Pacific Regional Office, Manila, The Philippines; 5 WHO Representative for the Solomon Islands, Honiara, Solomon Islands

**Keywords:** integrated primary care, Universal Health Coverage, Solomon Islands, primary healthcare policy

## Abstract

The Solomon Islands Government is pursuing integrated care with the goal of improving the quality of health service delivery to rural populations. Under the auspices of Universal Health Coverage, integrated service delivery packages were developed which defined the clinical and public health services that should be provided at different levels of the health system. The process of developing integrated service delivery packages helped to identify key policy decisions the government needed to make in order to improve service quality and efficiency. The integrated service delivery packages have instigated the revision of job descriptions and are feeding into the development of a human resource plan for health. They are also being used to guide infrastructure development and health system planning and should lead to better management of resources. The integrated service delivery packages have become a key tool to operationalise the government’s policy to move towards a more efficient, equitable, quality and sustainable health system.

## Introduction

Adhering to the guiding principle of Universal Health Coverage, the Solomon Islands’ Ministry of Health and Medical Services is pursuing *integrated care* with the goal of improving the range and quality of services available to the population in line with the concept of primary health care and with the overall vision of ‘health for all’, an ideal originally declared at the International Conference on Primary Health Care at Alma-Ata [1]. The World Health Organization Representative Office for the Solomon Islands has been supporting this process by facilitating the development of service delivery packages for primary health care that redefine which services should be available at each level of the health system and who should provide those services. The policy, once fully implemented, would represent a fundamental shift to people-centred health care, not to be mistaken for patient-centred health care, and aligns with the recent World Health Organization Global Strategy on people-centred and integrated health services [2]. These packages of services have become a key tool in the operationalisation of a change in primary healthcare policy in the Solomon Islands and this experience has provided valuable lessons to be learned for integrated care initiatives in similar settings such as low-resource and/or small island developing states.

Adhering to the guiding principle of Universal Health Coverage, the Solomon Islands’ Ministry of Health and Medical Services is pursuing *integrated care* with the goal of improving the range and quality of services available to the population in line with the concept of primary health care and with the overall vision of ‘health for all’, an ideal originally declared at the International Conference on Primary Health Care at Alma-Ata [1]. The World Health Organization Representative Office for the Solomon Islands has been supporting this process by facilitating the development of service delivery packages for primary health care that redefine which services should be available at each level of the health system and who should provide those services. The policy, once fully implemented, would represent a fundamental shift to people-centred health care, not to be mistaken for patient-centred health care, and aligns with the recent World Health Organization Global Strategy on people-centred and integrated health services [2]. These packages of services have become a key tool in the operationalisation of a change in primary healthcare policy in the Solomon Islands and this experience has provided valuable lessons to be learned for integrated care initiatives in similar settings such as low-resource and/or small island developing states.

## Background

The Solomon Islands Government is dealing with the challenge of serving widely dispersed and often remote island communities with serious shortages of health workers, essential drugs, clinical equipment and medical supplies [3]. The top 20 causes of morbidity and mortality indicate the country is dealing with the ‘double disease burden’ of both communicable and non-communicable diseases [4]. There is also an urgent need for upgrade, repair or renovation of around 345 health facilities in a country serving a population of only around 670,000 [5]. The economy is largely based on subsistence agriculture, and there are major concerns for sustainable development with the recent heavy reliance on unsustainable logging and high foreign aid dependency [4]. These factors, combined with high population growth, are likely to impact on the government’s ability to move towards their stated vision of a strong, affordable and efficient health system that improves population health status [6]. The government is aware of these issues and is acting to increase access to quality health services. The costs of achieving and sustaining uniform coverage of preventive and primary care services will, however, be difficult unless the efficiency of the health system can be improved to better utilise allocated resources.

Integration of the health system and coordination of primary health care have been identified as key strategies to increase the effectiveness of the health system in providing services to the population. Currently, the health system is vertically structured and managed from the Solomon Islands Ministry of Health and Medical Services headquarters in Honiara. Moving responsibility of programme implementation to the provinces will allow Ministry of Health and Medical Services to focus only on policy, strategy and monitoring and evaluation while Provincial Health Offices, who know their areas and populations best, will be better placed to provide coordinated, targeted, people-centred health care to local populations.

Currently, 60% of all health funding is held and spent by the central ministry and around 24% of the total health work-force is based at the National Referral Hospital in the capital, Honiara [3]. Within the environment that the health system has to function, there is a definite push to decentralise SIG services, responsibilities and accountability to the provinces. With around 80% of the population living in rural areas [4] and the realisation that centralised management of services has not resulted in better health outcomes, it makes sense for health resources to be decentralised to improve services for rural populations closer to where they live. However, until primary and secondary provincial health facilities are strengthened, people will continue to travel to the *National Referral Hospital* to access a perceived higher quality of health care which, consequently, necessitates the concentration of resources in Honiara [4].

In 2001, the Solomon Islands Government signed a cooperation agreement with Cuba which led to the students of the Solomon Islands being offered scholarships to study medicine in Cuba [7,8]. Over the next 5 years, 25 Cuban-trained doctors will return to the Solomon Islands each year which has posed questions about the capacity of the system to absorb them due to the limited positions available in the hospitals, the supervisory capacity to manage them all and the cost of paying them in the current fiscal environment [9]. Their return will have implications for the way healthcare services are provided as, for the first time in the Solomon Islands, it has been decided that there will be enough doctors for some to be placed into rural health facilities. For these doctors to be successful, new equipment and infrastructure as well as new management and supervisory structures will be needed in rural areas.

## Description of the policy development

In 2011, the Ministry of Health and Medical Services developed the Role Delineation Policy which defined the roles and responsibilities of the different types of health facilities that make up the Solomon Islands health system [4]. This policy reflected the government’s strategy of strengthening services to rural populations while responding to changes in demand for health services. This policy direction was further affirmed in 2013 when the Universal Health Care approach was adopted as the Solomon Islands Government’s main health sector strategy. The policy reclassified the five tiers of the health system into four:

- Rural Health Centre
- Area Health Centre
  ◦ AHC Level 1
  ◦ AHC Level 2
  ◦ Urban Health Centre
- General Hospital and
- National Referral Hospital,

with the lowest tier classification, *Nurse Aide Post*, being phased out. The clear delineation of the different structural levels had some initial identification of the required human resources, medical equipment and medicines as well as the need for improvements to infrastructure. However, these listings were of a generic nature and were not linked to the package of services to be provided at each level.

Therefore in 2014, the Ministry of Health and Medical Services in collaboration with development partners led by the local World Health Organization country office embarked on a process to develop ‘integrated service delivery packages’ that would specify an essential package of services to be delivered at each level of the health system and the staffing, drugs, equipment and infrastructure required at the different types of facilities to provide those services. The integrated service delivery packages were developed through an iterative consultation process beginning with a workshop involving all stakeholders. National programme directors and staff then defined the services for their programme by facility level including *Rural Health Centre, Area Health Centre* and *General Hospital*. These individual packages reflected the strategic direction of each programme and was an approach led by service delivery as opposed to the initial Role Delineation Policy which obscured the centrality of service delivery. Tables 1,2,3,4 are extracts from the integrated service delivery packages detailing the level at which maternal and newborn care services will be provided. Once the services were identified, policy makers were also able to identify exactly which resources would be needed at each level.

**Table 1:** Family Planning services extract from the Integrated Service Delivery Packages.

			AHC			
				
	CMTY	RHC	L1	L2	UHC	GH

Education, counselling and motivation to adopt appropriate family planning methods	X	X	X	X	X	X
Provision of hormonal and barrier family planning methods	X	X	X	X	X	X
Birth planning	X	X	X	X	X	X
IUCD insertion			X	X	X	X
Non-scalpel vasectomy and bilateral tubal ligation				X	X	X

CMTY = Community, RHC = Rural Health Centre, AHC = Area Health Centre, L1 = Level 1, L2 = Level 2, UHC = Urban Health Centre, GH = General Hospital.

**Table 2:** Antenatal care services extract from the Integrated Service Delivery Packages.

			AHC			
				
	CMTY	RHC	L1	L2	UHC	GH

Birth and emergency planning	X	X	X	X	X	X
*Screening*: History taking, physical examination, HB, syphilis test, urinalysis, malaria RDT, blood pressure, weight		X	X	X	X	X
Respond to problems (observed and/or reported)		X	X	X	X	X
*Preventative measures*: deworming, tetanus toxoid vaccination, iron and folic acid tablets, malaria prophylaxis and long-lasting insecticide-treated bednet distribution		X	X	X	X	X
Stabilisation and referral of complicated pregnancies such as pre-eclampsia and breech		X	X	X	X	X
Treatment of maternal anaemia and infection		X	X	X	X	X
Ultrasound services				X		X

CMTY = Community, RHC = Rural Health Centre, AHC = Area Health Centre, L1 = Level 1, L2 = Level 2, UHC = Urban Health Centre, GH = General Hospital.

**Table 3:** Intra-Partum Care services extract from the Integrated Service Delivery Packages.

			AHC			
				
	CMTY	RHC	L1	L2	UHC	GH

Skilled care during childbirth for clean and safe normal delivery		X	X	X	X	X
Labour monitoring with obstetric wheel		X	X	X	X	X
*Essential newborn care*: basic newborn resuscitation + immediate and thorough drying + warmth (Kandora Mother Care) + eye prophylaxis + clean cord care + early and exclusive breastfeeding		X	X	X	X	X
*Basic emergency obstetric care*: parenteral antibiotics + oxytocic/anticonvulsant drugs + manual removal of placenta + removal of retained products with manual vacuum aspiration + assisted vaginal delivery *24/24 7/7*		X	X	X	X	X
Appropriate and prompt referral for cases needing specialised care		X	X	X	X	X
Stabilisation and referral of obstetric emergencies		X	X	X	X	X
IEC on nutrition, hygiene, contraception and essential newborn care		X	X	X	X	X
All routine birth dose immunisations and vitamins		X	X	X	X	X
Ventouse deliveries			X	X	X	X
Midwife management of labour			X	X	X	X
Augmentation of labour with medical indication						X
*Comprehensive emergency obstetric care*: basic emergency obstetric care + caesarean section + safe blood transfusion						X
Presumptive antibiotic therapy for newborns at risk of bacterial infection				X	X	X
Continuous positive airway pressure to manage babies with respiratory distress syndrome						X
Case management of neonatal sepsis, meningitis and pneumonia				X	X	X
Detection and management of postpartum sepsis				X	X	X

CMTY = Community, RHC = Rural Health Centre, AHC = Area Health Centre, L1 = Level 1, L2 = Level 2, UHC = Urban Health Centre, GH = General Hospital.

**Table 4:** Post-Partum Care services extract from the Integrated Service Delivery Packages.

			AHC			
				
	CMTY	RHC	L1	L2	UHC	GH

Examination of mother and newborn (up to 6 weeks)	X	X	X	X	X	X
Respond to observed signs	X	X	X	X	X	X
Support breastfeeding	X	X	X	X	X	X
Promote family planning	X	X	X	X	X	X

CMTY = Community, RHC = Rural Health Centre, AHC = Area Health Centre, L1 = Level 1, L2 = Level 2, UHC = Urban Health Centre, GH = General Hospital.

**Figure 1:**
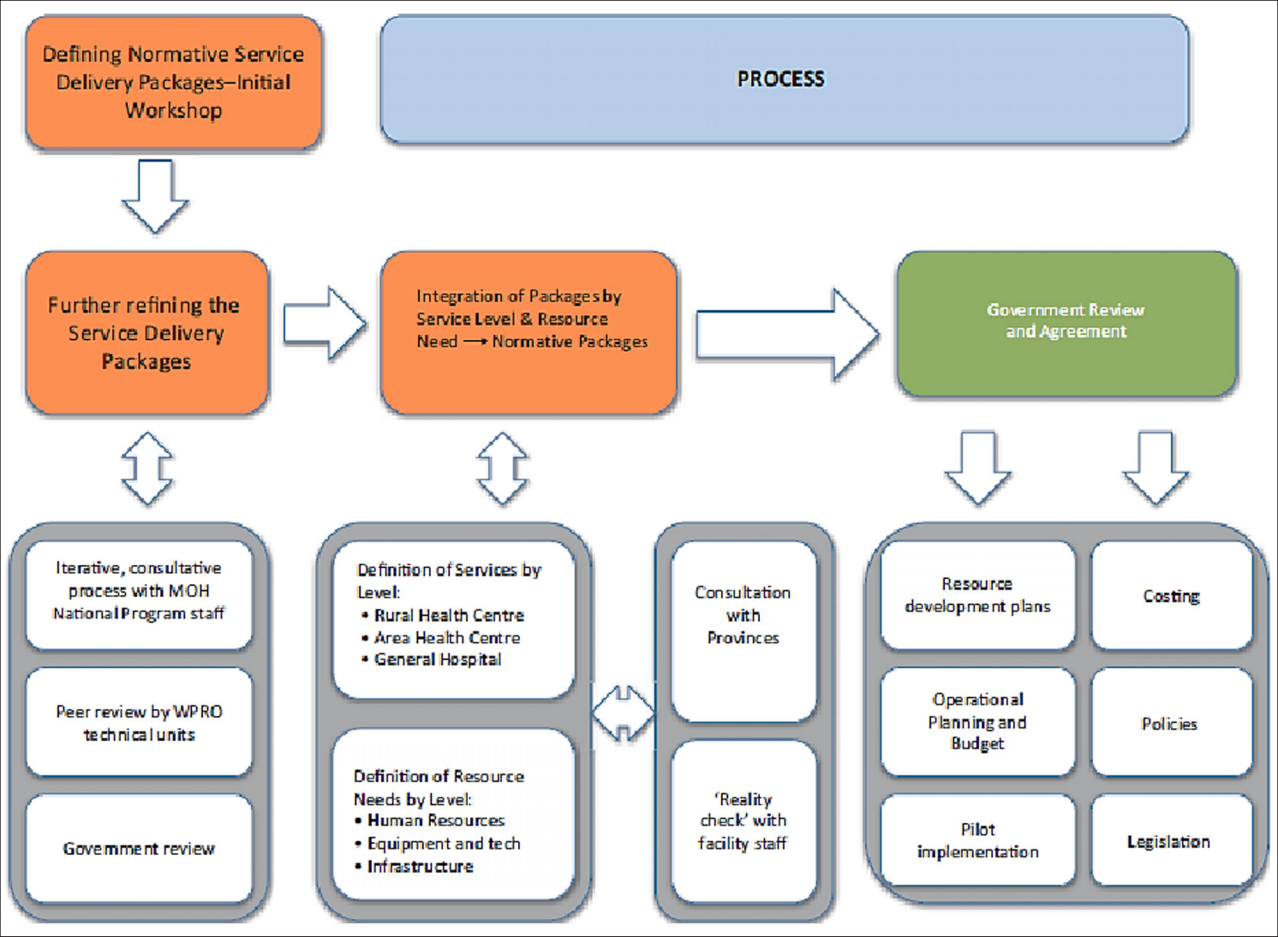
Integrated service delivery policy development.

Technical content of the individual packages was internationally peer reviewed by experts from World Health Organization and other development partners before being integrated by facility type, resulting in comprehensive packages of services which reflected the strategic direction of all national health programmes. Synergies between programmes in terms of services and resources were identified as well as potential areas for efficiency gains. Subsequently, a secondary process was instigated through consultations with both clinical and public health staff from the national, provincial and community level to further refine the packages while identifying the requirements for implementation and making adjustments to ensure they were implementable. The latter process was also used to define the role of each type of health facility and link the different facilities and levels together as a functional system. However, the process of development highlighted a number of challenges for moving towards the government’s stated vision of a strong, affordable and efficient health system that improves population health status [6]. Figure 1 summarises this process as well as the next steps.

## Discussion

The development of the integrated service delivery packages highlighted a major disconnect between the expectations of national programme directors and the capacity of the available workforce. In the Solomon Islands, almost all health workers based at *Rural Health Centre* and *Area Health Centre* level are nurses or nurse aides and there are currently no doctors outside the hospitals. With this reality and the existing disease burden, it was clear that a move to a multi-disciplinary team model at *Area Health Centre* was needed for uniform coverage of the integrated service delivery packages to be achievable. The current situation where nurses are responsible for providing all services in rural health facilities will need to change to enable the delivery of essential services as defined by national policy makers.

To shift from the current nurse-based model to a multi-disciplinary model, Ministry of Health and Medical Services is exploring possibilities for better utilisation of nurse aides to perform their primary role in the community while developing multi-skilled clinical and public health staff to perform a number of roles at *Area Health Centre* level. For example, a job description for a generalist *Public Health Officer* has been developed at *Area Health Centre* level whose main responsibility will be the monitoring and implementation of the ‘Healthy Settings’ strategy, a holistic and multi-disciplinary health promotion approach that integrates action across risk factors, and is a key component of the integrated service delivery packages. While exploring how to implement the integrated service delivery packages efficiently, it was agreed that this officer could report to multiple public health programmes at the provincial level such as the environmental health, community-based rehabilitation and social welfare managers so that they are better informed on issues in the community and are able to plan and implement targeted activities. This is one example of how the integrated service delivery packages can have a range of potential benefits and the revision of job descriptions for health workers is ongoing. It is also anticipated that the integrated service delivery packages will feed into the development of the first human resource plan for health and the modification of training programmes for health workers.

A lack of human resource planning has led to the situation where more doctors than are needed have been sent to Cuba for training and who will be returning to the Solomon Islands to work over the next 5 years. The development of the integrated service delivery packages and the current fiscal environment has prompted discussions as to how they can be most effectively utilised to ensure the best outcomes for population health. The training these student doctors are receiving in Cuba focuses on primary care and community medicine, so they should, in theory, be most effective when placed in rural health facilities. A secondary benefit is that Area Health Supervisors, usually the clinical nurse based at the *Area Health Centre* who is responsible for clinical and administrative supervision and management of all staff in the zone, will be able to focus on their administrative duties due to the presence of the doctor who can provide the clinical leadership. Through the consultation process described above, Area Health Supervisors reported that their current clinical workload was a barrier that prevented organising regular outreach visits to the community, collecting and collating data from *Rural Health Centres* and adequately monitoring equipment and infrastructure maintenance needs in the zone. This is just one example of a number of potential benefits of placing a doctor at the *Area Health Centre* level to provide the clinical leadership and support needed.

Discussions around the re-classification of the levels of the health system and the phasing out of the lowest level of health facility raised some key decisions that the government needed to make. With over 345 health facilities serving a population of around 670,000, there are a high number of health facilities for the population and a number which the government struggle to maintain and adequately resource. Assessments of health facilities in the Solomon Islands found that many were operating without proper water and sanitation, electricity and basic medical supplies [10,11,12].

The degradation of health facilities has happened over time mainly due to a lack of funding from the national government and poor budgeting preventing adequate maintenance [10]. The development of the integrated service delivery packages and the reality of how difficult it will be to achieve universal coverage of these services with the current service configuration has helped to identify that a shift in priorities is needed: from the current focus on increasing coverage through construction of new facilities which end up being ill-equipped to provide quality health services, to a focus on rationalising the current facilities and increasing coverage of a package of *quality* health services. Outreach visits to provide preventive services in the community from a well-equipped and staffed facility will be a crucial component of this strategy.

There are ongoing discussions on how to resolve the current issue where Ministry of Health and Medical Services does not always decide where and when a health facility is constructed. Facilities are often built by communities and local politicians which Ministry of Health and Medical Services then need to staff and resource. This is one urgent issue that is yet to be resolved, but the development of a *partner coordination* unit which reports to the Ministry of Health and Medical Services executive is planned to address this. Eventually, a licensing or facility registration system that ensures new health facilities are only constructed in line with Ministry of Health and Medical Services planning processes is needed to ensure the sustainability of the health system.

As the current workforce is distributed across a large number of facilities, outreach activity such as maternal and child health services provided through village and school visits and basic preventative health services to remote areas in general are very limited. The strengthening of integrated outreach visits to villages and schools, involving multi-disciplinary primary healthcare teams to deliver services and support the implementation of the *Healthy Settings* policy, is being explored as a more efficient way to provide services to remote areas rather than building new physical facilities.

The strategy for increasing outreach activities is through quantifying the number of outreach visits required each year and by developing a standard for what services should be provided through outreach. As delivery of specialist outreach services is currently dependent on the skill mix and workload of nurses at a particular location, an increase in outreach activities will also be assisted by better human resource planning as well as the expansion of staffing at *Area Health Centre* level to include multi-disciplinary clinical and public health staff who have the capacity to coordinate, support and supervise the preventative health activities of facilities within the zone.

Implementation of the integrated service delivery packages will coincide with the horizontal integration of processes at the provincial level along with the decentralisation of resources. In the Solomon Islands, most Provincial Health Directors are clinicians and spend a significant proportion of their time providing clinical services in addition to their role in the actual planning and management of health services. Provincial Health Offices will need to take ownership of policy implementation, so it is crucial that they have the capacity and support to be able to manage the increased responsibility. For this reason, there are proposed changes to the provincial management structure and reporting lines to ensure Provincial Health Directors are able to efficiently manage their own resources. One example is a new Director of Corporate Services position at the provincial level so that the province can handle the additional administrative workload. Adapting and streamlining the existing vertical or ‘silo’ health system structure in the Solomon Islands, complementing the decentralisation of health resource management to the provinces, will be key to the successful implementation of the integrated service delivery packages.

## Conclusion

The integrated service delivery packages have become a key tool to drive the changes made necessary by the government’s adoption of the Role Delineation Policy. First and foremost, the definition of service roles will enable the equitable allocation of resources and adequate support and supervision of lower levels of care. However, it will also be used to develop referral and coordinated delivery arrangements as well as new provincial management structures and reporting lines. The process of development has been highly valuable in identifying gaps in the health system as well as the key challenges and policy decisions that need to be addressed to enable the strengthening of services to rural populations. At the *Area Health Centre* level, where there are major opportunities for improvements in quality and efficiency through integration of service delivery, pilot implementation of the integrated service delivery packages is underway. Evaluation of these trials will be used to refine the integrated service delivery packages and continue to inform changes to the service delivery model.

The consultation process described above was crucial to gaining the support of national and provincial stakeholders who would be responsible for implementing the integrated service delivery packages. It is important that local leaders understand the policy and are able to advocate for it so that they can build support. Developing peripheral management capacity will be critical to the success of the decentralisation agenda.

Striving for people-centred health care should guide any integrated care initiative, and World Health Organization have recently developed a Global Strategy that will assist its mission statement to achieve Universal Health Cover-age with more people-centred and integrated health services [2]. In this case, by first agreeing on the services that should be available to rural populations and then working out how that would be achieved, it was possible to identify and look for ways to overcome the barriers to providing those services.

Development of the integrated service delivery packages helped to identify that an improvement in both service quality and efficiency would require the existing vertical structure in the health system, where individual programmes predominantly function independently of each other, to be better integrated at the national and provincial level. Increasing service quality and efficiency may also be helped by better coordination of primary health care through decentralising responsibility and capacity for programme implementation to the provincial level. With effective management and leadership, a decentralised health system that is more flexible and responsive to local population health needs should be more effective at targeting limited resources where they are needed.

The process of developing integrated service delivery packages for the Solomon Islands can provide lessons for health policy makers as it was the process itself that was key to identifying a number of inefficiencies in the health system, barriers to improving service quality and potential costs to the health system which needed to be mitigated against. The process highlighted a number of key policy decisions that needed to be made and helped health lea-ders to formulate a strategy to move towards a more efficient, equitable, quality and sustainable health system. It is hoped that when resources, staff, information and service interventions are sufficient and work in unison, there will be an improvement in population health outcomes.

## Competing Interests

The authors declare that they have no competing interests.

## Reviewers

**Lucinda Cash-Gibson**, Researcher, Universitat Pompeu Fabra, Barcelona, Spain.

**Jason CH Yap**, Associate Professor, Saw Swee Hock School of Public Health, National University of Singapore, Singapore.

Two anonymous reviewers.
